# Quality of life following surgical repair of acute type A aortic dissection: a systematic review

**DOI:** 10.1186/s13019-022-01875-x

**Published:** 2022-05-16

**Authors:** Aditya Eranki, Ashley Wilson-Smith, Michael L. Williams, Akshat Saxena, Ross Mejia

**Affiliations:** 1 Department of Cardiothoracic Surgery, John Hunter Hospital, Newcastle, NSW 2305 Australia; 2 The Collaborative Research (CORE) Group, Macquarie University, Sydney, Australia; 3 School of Medicine and Public Health, University of Newcastle, Newcastle, Australia; 4 Department of Cardiothoracic Surgery, Fiona Stanley Hospital, Perth, Australia

**Keywords:** Health related quality of life, Type A aortic dissection, Outcomes, Systematic review

## Abstract

**Background:**

The outcomes of surgery for acute Stanford Type A aortic dissection (ATAAD) extend beyond mortality and morbidity. The aim of this systematic review was to summarise the literature surrounding health related quality of life (HR-QOL) following ATAAD, compare the outcomes to the standardised population, and to assess the impact of advanced age on HRQOL outcomes following surgery.

**Methods:**

A systematic review of studies after January 2000 was performed to identify HR-QOL in patients following surgery for ATAAD. Electronic searches of three databases were performed and clinical studies extracted by two independent reviewers. Strict inclusion and exclusion criteria were applied. Quality appraisal was conducted utilizing predefined criteria on pilot forms. HR-QOL results were synthesized through a narrative review of included studies.

**Results:**

There was significant attrition in HR-QOL of patients following surgery for ATAAD. Outcomes fared worse when compared to an age adjusted normative population. Of note, elderly patients were physically vulnerable, whereas younger populations may be more mentally vulnerable to postoperative sequalae. The included studies were quite heterogeneous in their study designs, methods, HR-QOL measures reported and follow up time-frames which limited direct comparison between studies.

**Conclusion:**

HR-QOL outcomes are adversely affected when compared to preoperative status and physical health demonstrates significant attrition over time. HR-QOL outcomes are worse off when compared to an age matched general population. In terms of age, advancing age is associated with worse physical component scores but emotional health may fare better than younger patients.

**Supplementary Information:**

The online version contains supplementary material available at 10.1186/s13019-022-01875-x.

## Introduction

Acute Type A aortic dissection (ATAAD) represents a cardiothoracic emergency. Conventional wisdom dictates that mortality rate increases 1–2% per hour within the initial 48 h, with the postoperative 30-day mortality varying between 10 and 35% [1, 2]. ATAAD is additionally associated with a high morbidity rate, with a range of postoperative sequelae such as stroke, prolonged intubation, myocardial ischemia, mesenteric ischemia, limb ischemia and renal failure [3–5]. Long-term data suggests that the 10-year survival rate post-surgery is 50% [6–8].

The outcomes of surgery for ATAAD extend beyond mortality and morbidity rates. Postoperative health-related quality of life (HR-QOL) provides information on the physical, mental, emotional and functional well-being of patients following surgery. HR-QOL research is well validated within cardiac surgery, with the Short Form 36 (SF36) being the most common questionnaire [9, 10]. Existing systematic reviews suggest positive HR-QOL outcomes following cardiac surgery [11, 12]. Evidence is accumulating that demonstrates diminished quality of life after surgery for ATAAD [13–29]. Of note, elderly patients demonstrate a significantly higher mortality rate and lower quality of life following surgery.

This systematic review aims to summarise the literature surrounding HR-QOL following surgery for ATAAD, compare the outcomes to the standardised population, and assess the impact of age on HR-QOL outcomes following surgery.

## Methods

### Literature search strategy

**Fig. 1:**
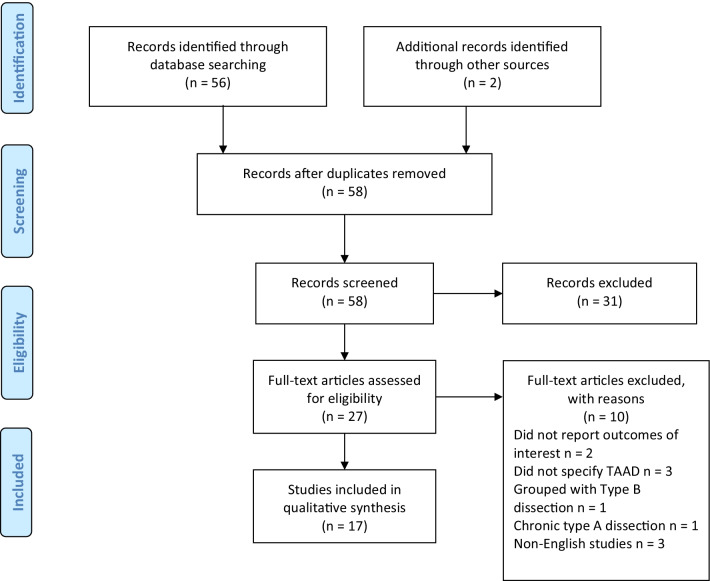
PRISMA flow-chart summarizing the search strategy for relevant publications

This systematic review was performed in line with Preferred Reporting Items for Systematic Reviews and Meta-Analyses (PRIMSA) recommendations and guidance [30, 31]. An electronic literature search was conducted using PubMed, EMBASE and MEDLINE databases from January 2000 to the 16th of July 2021. The search strategy included a combination of keywords and Medical Subject Headings (MeSH) including “health related quality of life” AND “Stanford Type A Aortic Dissection”, NOT “Stanford Type B Aortic Dissection”. The search strategy is outlined in Fig. 1. Additionally, references of each included study were assessed to identify any extra relevant studies. Following the search, two investigators (A.E and A.W.S) independently performed the first stage of screening titles and abstracts. Studies were excluded if they did not meet the eligibility criteria. If the title and/or abstract contained material that met eligibility criteria, the full-text article was retrieved to further assess if the article met eligibility criteria. Studies were excluded if they did not meet eligibility criteria for various reasons. Consensus for study inclusion was achieved by discussion between two investigators (A.E and A.W.S).

### Inclusion and exclusion criteria

Studies were considered eligible if they demonstrated (1) Patients undergoing emergent surgery for ATAAD (2) published past the year 2000 (3) HR-QOL measures were clearly reported for patients who underwent surgery for ATAAD, and (4) published in English. Studies were excluded if they did not meet inclusion criteria.

### Study quality appraisal

Quality appraisal was conducted by two independent reviewers (A.E and MW). Study quality was assessed with the Delphi Study Quality Appraisal tool (Additional file 1: Table S1) [32]. Focus was paid to study design, number of included patients, clear inclusion of patients with ATAAD, standardised HR-QOL measure, whether the primary aim of the paper was to report HR-QOL, comparison with an age matched cohort, comparison to preoperative status and adequate follow up rate and timeframe.

### Data extraction and statistical analysis

Data was extracted using a pilot form. Demographic details including mean age, gender, smoking status, diabetes and operative details were extracted Long term survival from Kaplan Meier curves was digitized where presented and an algorithmic computational tool was utilised to derive individual patient data, and an algorithmic tool was utilised as outlined by *Guyot et al* [33]*.* Event and censoring data was compiled for 5 years, and overall survival curves were produced utilising SPSS version 26 (Armonk, New York, United States of America). Qualitative analysis of HR-QOL data was performed, by extracting data from each study using a standardised pilot form. Studies were then grouped with regards to variable of interest and summarised in a series of tables below (Tables 3, 4, 5). Due to study heterogeneity in methodology and reporting of outcomes, statistical analysis was not possible for HR-QOL outcomes.

### Definitions

The World Health Organization (WHO) defines HR-QOL as an individual’s perception of their position in life in the context of the culture and value systems that they live in, and in relation to their goals, expectations, standards and concerns [34]. It can act as a tool for determining the true effectiveness of a procedure and assist in driving healthcare policies [35]. A standardised measurement is required that is validated, reproducible and covers all domains [36].

A number of HR-QOL measures are validated in cardiac surgery. Of these, the Short Form 36 (SF36) is most widely used [10]. It consists of 36 individual items, grouped into eight scales. Collectively, the eight scales can be collated into two higher order domains representing physical component scores (PCS) and mental component scores (MCS) of QOL [10]. The SF36 has also been used to demonstrate HRQOL outcomes in elderly patients [10]. It is yet to be validated in the area of aortic surgery, however, is still widely applied. Other validated measures of HR-QOL include the EuroQol-5D (EQ5D) and the Patient-Reported Outcomes Measurement Information System (PROMIS), both of which are robust in the setting of cardiac surgery [36, 37]. These are summarised in Table 1.

**Table 1:** HR-QOL measurement

HR-QOL Measure	Domains	Description
SF12/SF36 [38]	36 (or 12) items measuring 8 conceptual domains or dimensions of health [36]	
PCS	Physical functioning (PF)	Limitations of physical activity including walking and dressing
	Role-physical (RF)	Limitations of regular daily activities because of physical health
	Bodily pain (BP)	Amount of pain and interference with regular daily activities
	General health (GH)	Rating of health, comparison with others
MCS	Vitality (VT)	Energy and tiredness ratings
	Social functioning (SF)	Limitations to time and type of social activities
	Role-emotional (RE)	Limitations of regular daily activities because of emotional problems
	Mental health (MH)	Anxiety/serenity depressed mood/happiness
EQ5D [39]	five dimensions (mobility, self-care, usual activities, pain/discomfort, anxiety/depression) each with three or five response levels [37]	
	Mobility	Ability to walk
	Self care	Ability to dress
	Usual activities	Activities of daily living
	Pain/Discomfort	Level of pain
	Anxiety/Depression	Level of anxiety or depression
PROMIS [40]	A set of patient -centred measures that evaluates and monitors physical, mental and social health of adults	

## Results

The literature search identified 54 studies. An additional two articles were identified on manual searches of reference lists. After exclusion of irrelevant studies, 27 articles were deemed appropriate to undergo full-text review. Seventeen studies with a total of 2,388 patients were deemed suitable for inclusion for qualitative analysis in this review [13–29]. Three studies were prospective, and the remaining 14 were retrospective cohort studies. Included studies had varying final cohorts from 12 to 210 patients. The majority of studies assessed all age groups and four studies assessed HR-QOL in the elderly. These results along with study quality is summarised in Additional file 1: Table S2.

### HR-QOL measurements

The majority of studies utilised the SF36 or SF12 as a primary HR-QOL measurement [13–21, 25, 26, 28]. One study utilised the EQ5D scale [22]. One utilised the PROMIS questionnaire [27]. Studies assessed other parameters of well-being including VO2 max, sexual function, post traumatic stress disorder (PTSD) scales or derived their own HR-QOL questionnaire [13, 21, 23, 24, 27, 29].

### Baseline data

The mean age of patients ranged from 53 to 78 years old. The majority of patients were men, ranging from 33 to 86%. Tashima et al., reported the lowest incidence of preoperative neurological deficit at 6%, and Adam et al. reported the highest at 41%. Preoperative malperfusion was reported in 10 studies, and varied from 18 to 57%. Most commonly, patients underwent a replacement of the ascending aorta (ranging from 66 to 100%). Aortic arch replacement was performed variably, ranging from 0 to 33%. Reporting of deep hypothermic circulatory arrest (DHCA) and open distal anastomoses was variably reported. The use of DHCA varied from 52 to 100% (in 7 studies). Similarly, cerebral perfusion strategies were variably reported. The most common was selective antegrade cerebral perfusion (ACP), which varied from 26 to 100% (in three studies). The use of retrograde cerebral perfusion was reported in four studies. Baseline data is summarised in Additional file 1: Table S3.

**Fig. 2:**
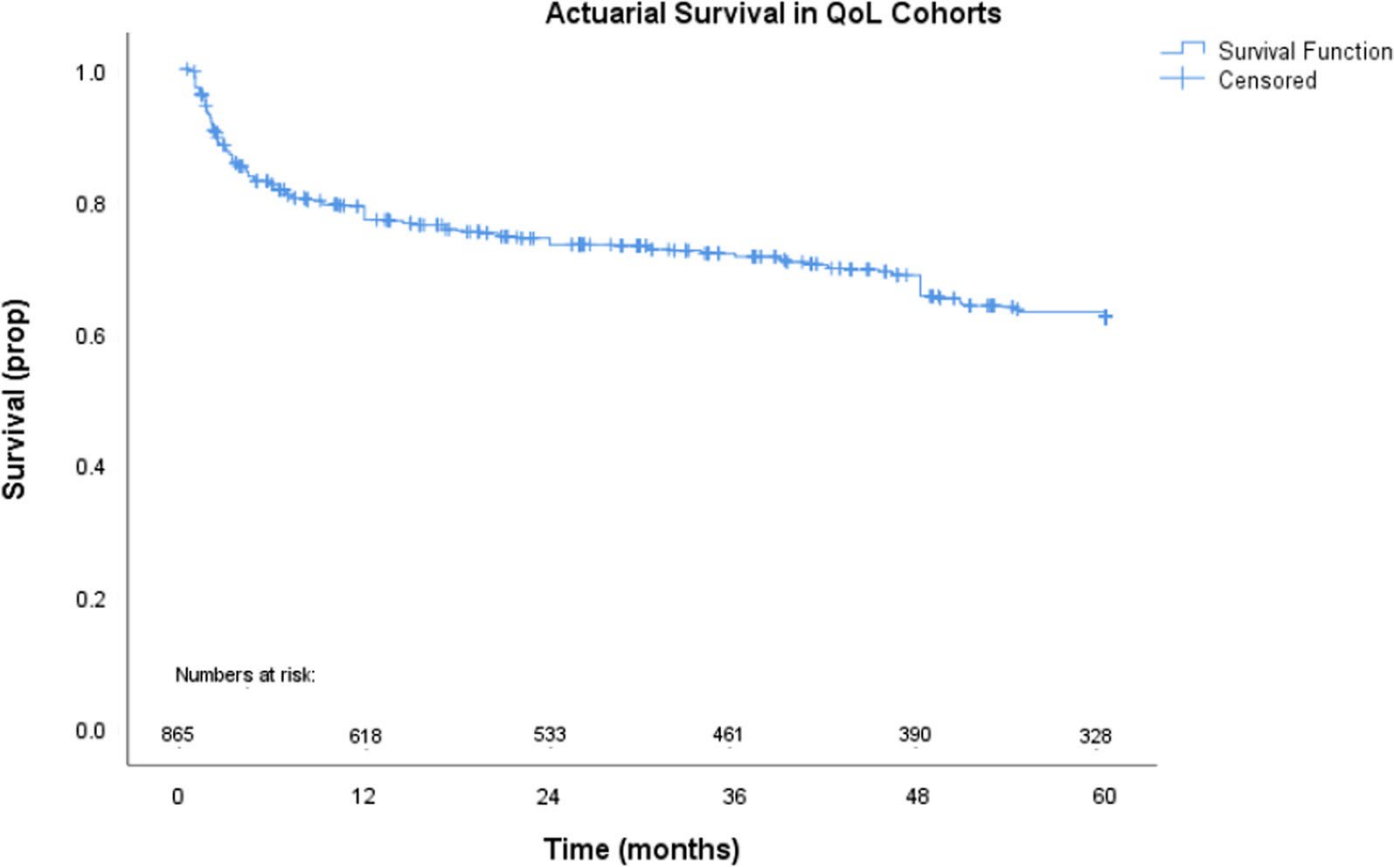
Aggregated overall survival after ATAAD from included studies

Short-term mortality was reported in 12 studies. This ranged from 8.9% to 40%. Six studies reported data in an elderly cohort of patients and the mortality rate ranged from 0 to 40%. Mortality data is summarised in Table 2. Long-term mortality was reported in eight studies. Aggregate actuarial survival at 1-, 2-, 3-, 4- and 5-years was 79%, 73%, 72%, 69% and 62% respectively (Fig. 2.).

**Table 2:** 30-day Mortality in surgically treated ATAAD

Study	Study population	Mortality
Endlich et al	All patients	17.40%
Bojko et al	Octogenarians and Septuagenarians	Overall 23.4%
		28.6% > 80Y
		21.2% > 70Y
St Pierre et al	All patients	8.90%
Santini et al	Patients > 75Y	30%
Jussli-Melchers et al	Septuagenarians vs non Septuagenarians	Overall 12.4%
		7.9% < 70
		21.8% > 70
Tang et al	Octogenarians and non octogenarians	Overall 9%
		0% > 80
		11% < 80
Schachner et al	All patients	13.50%
Van Huyse et al	Octogenarians and non octogenarians	Overall 19.5%
		40% > 80Y
		18% < 80Y
Sbarouni et al.	All patients	24%
Campbell Lloyd et al	All patients	23.53%
Tashima et al	Septuagenarians vs non Septuagenarians	Overall 7.8%
		11.8% < 70Y
		3.8% > 70Y
Immer et al	ATAAD patients who underwent DHCA arrest	15.90%

*Y* years old

### HR-QOL vs time

Seven studies reported the change on HR-QOL measures of patients over time [14, 16, 23–25, 27, 29]. Four studies reported the change of HR-QOL preoperatively (prior to the ATAAD) and post operatively [16, 23, 24, 29]. Three of the included studies followed patients purely over the postoperative period [14, 25, 27]. Five studies reported a worse of HR-QOL over time [14, 16, 24, 27, 29]. One study noted that at best, HR-QOL did not demonstrate significant attrition over time postoperatively [25]. Results for HR-QOL over time are summarised in Table 3.

**Table 3:** HR-QOL in ATAAD over time

Study	HR-QOL measurement	Follow up timeframe	HR-QOL outcomes
Endlich et al. [14]	SF-12	Postoperatively: early (45 months) late (46 months)	PCS was significantly lower across two follow ups: PCS1 43.2 ± 11.0 vs PCS2 38.4 ± 9.9
			MCS was significantly lower across two follow ups: MCS1 48.1 ± 11.9 vs MCSII 33.3 ± 11.9
St Pierre et al. [16]	SF-36	Pre and postoperatively	PCS, was significantly lower post surgery compared to pre surgery (39 vs 49)
			MCS, pre and post surgery scores were not significantly different (50 vs 49)
			Patients scored significantly lower post surgery in all domains except mental health
Schachner et al. [23]	Self reported questionnaire	Pre and postoperatively	An equal number of patients were physically active pre and postoperatively
Van Huyse et al. [24]	WHO performance status scale	Pre and postoperatively	Preoperatively: 12 patients scored 0 or 1 for performance status, three patients scored 2
			Postoperatively: one patient scored 3 for performance status, six patients scored 2, and two patients scored 1
Sbarouni et al. [25]	SF-36	Postoperatively at 1, 5, and 10 years	PCS scores were significantly higher at 5 years than 1 and 10 years (50.3 vs 45.4 and 46.8 respectively *P* = 0.008)
			MCS scores at 5 and 10 years were comparable and significantly higher than at 1 year (49.7 and 49.1 vs 42.8 respectively *P* = 0.001)
Norton et al. [27]	PROMIS	Postoperatively: early (3 months) and late (15 months)	Thirty-seven percent of participants reported moderate-to severe anxiety at the early time point compared to 16% at the late time-point (*P* = 0.0061)
Tashima et al. [29]	Barthel index	Pre and postoperatively	Preoperative Barthel index 96.6, 91.3% walking without aids
			Postoperative: Barthel index 79.4, 50.5% walking without aids

*HR-QOL* health related quality of life, *SF* short form, *WHO* World Health Organisation, *PROMIS* patient-reported outcomes measurement information system, *MCS* mental component score, *PCS* physical component score

### HR-QOL vs normative values

Seven studies reported HR-QOL measures with reference to an age matched cohort [13, 14, 18, 19, 25, 26, 28]. Four studies demonstrate worse HR-QOL outcomes when compared to an age matched cohort [13, 14, 19, 26]. No studies report an improvement in HR-QOL. HR-QOL vs normative values data are summarised in Table 4

**Table 4:** HR-QOL in ATAAD vs age adjusted normative samples

Study	HR-QOL measurement	Result
Adam et al. [13]	SF12	The mean PCS for the ATAAD group were significantly lower than the norm sample (37.2 ± 10.9 vs 48.2 ± 8.8, *P* < 0.001)
		The mean MCS was for the ATAAD group was significantly lower than the norm sample 48.9 ± 11.6 vs 51.4 ± 8.6, *P* < 0.001
Endlich et al. [14]	SF36	PCS1 43.2 ± 11 and PCS2 38.4 ± 9.9 were significantly lower in the ATAAD group than the norm *P* = 0.001
		MCS1 48.1 ± 11.9 and MCS2 33.3 ± 11.9 were significantly lower in the ATAAD group than the norm *P* = 0.001
Santini et al. [18]	SF36	There were no significant differences between the ATAAD group and the norm sample in all SF36 domains
Jussli-Melchers et al. [19]	SF36	Pain scores, role limitations due to physical health, social functioning, role limitation due to emotional health and emotional well-being were significantly lower in the ATAAD group than the normative sample (*P* < 0.05)
		Overall PCS and MCS scores did not vary significantly between the ATAAD groups and the normative sample
Sbarouni et al. [25]	SF36	PCS and MCS scores at the 10 year follow up were comparable to the normative sample
Immer et al. [26]	SF36	Compared to the normative sample, Physical functioning, Role functioning physical and general health scores were substantially lower
Olsson et al. [28]	SF36	There were no significant differences in MCS and PCS scores when compared to the norm (44 vs 45 and 48 vs 50 respectively)

*HR-QOL* health related quality of life, *SF* short form, *PCS* physical component score, *MCS* mental component score, *ATAAD* acute type A aortic dissection

### HR-QOL in the elderly

Eight studies reported HR-QOL measures in the elderly (Table 5) [13–15, 18–20, 24, 29]. Five studies report worse of HR-QOL outcomes in the elderly [13, 14, 20, 24, 29]. Of note PCS were significantly worse in this demographic [13, 14, 20]. Some studies suggest that MCS are worse in younger cohorts [14, 20].

**Table 5:** HRQOL in elderly patients with ATAAD

Study	HR-QOL measurement	Patient Cohort	Results
Adam et al. [13]	SF12	Age by decade	PCS scores were significantly lower than the norm in the > 70 cohort. PCS scores decline with increasing age
			MCS scores were not significantly lower in the > 70 cohort compared to a normative sample
Endlich et al. [14]	SF36	Age by decade	PCS scores were significantly lower than the norm for > 70 cohort (36 vs 41.8 *P* = 0.015)
			MCS scores were significantly lower than the norm for > 70 (38.9 vs 52.1, *P* = 0.001)
			The younger the patient, the lower the MCS score
Bojko et al. [15]	SF36	Octogenarians vs septuagenarians	There were no significant differences in the distribution of responses between octogenarians and septuagenarians for any of the 36 questions
Santini et al. [18]	SF 36	Patients > 75Y	There were no significant differences between the SF36 domains between the study group and the general Italian population > 75 years
Jussli-Melchers et al. [19]	EQ5D	Patients > 70Y vs patients < 70Y	There were no significant differences between the younger and older groups across all domains
Tang et al. [20]	SF36	Patients > 80Y vs patients < 80Y	Physical functioning was significantly worse in the older group (43 vs 62, *P* < 0.05), whereas role limitations due to emotional health were less frequent in the older group (87 vs 66 *P* < 0.05)
			Other parameters were similar between groups
Van Huyse et al. [24]	WHO performance scale	Patients > 80	Six patients were able to return home postoperatively (40%). Every patient had a decrease in performance status level postoperatively
Tashima et al. [29]	Barthel index	Patients > 70Y vs patients < 70Y	There was a significant difference in the Barthel index at discharge between the over 70/under 70 cohorts (84.7 vs 74.5 respectively)

*HR-QOL* health related quality of life, *SF* short form, *PCS* physical component score, *MCS* mental component score, *ATAAD* acute type A aortic dissection

## Discussion

Quality of life is a useful indicator of overall health as it captures information on the physical and mental health status of a patient. This provides a comprehensive assessment of the burden of disease. Evidence is mounting that HR-QOL outcomes are under utilised and have merit in assessing outcomes in cardiac surgical patients [10]. There is a paucity of literature assessing HR-QOL outcomes in patients who have undergone surgical repair of ATAAD [10].

Study heterogeneity and variability of reporting prevented direct comparison of results. The primary outcome of four studies was to report patients undergoing surgery for thoracic aortic aneurysm with aortic dissection being a subset of patients included in the analysis [16, 26–28]. Three studies exclusively assessed elderly patients undergoing surgery for ATAAD [15, 18, 24]. HR-QOL outcomes were also variably reported. The majority of studies utilised SF-36 or SF-12 [13–21, 25, 26, 28]. Within this, there was still variability with reporting of SF domains, with some papers utilizing MCS and PCS scores whereas others reporting individual domains. One of the included studies utilised the EQ-5D scale [22] whilst another utilised PROMIS [27]. Three included studies utilised a non-standardised HR-QOL outcomes, which makes direct comparison to standardised cohorts unattenable [23, 24, 29]. Baseline variables were also variably reported, with a number of studies not reporting operative practices, the use of DHCA or cerebral perfusion strategies. These may have an impact on postoperative QOL. The variability of reporting and differences in baseline variables was also a limiting factor for meta-analysis.

The majority of studies were retrospective with only three studies being prospective [14, 17, 27]. The retrospective design has inherent bias and contributes to a lack of data on whether these patients improved from their preoperative state and by what magnitude. Retrospective design also introduces recall bias, reducing the integrity of patient responses.

The HR-QOL over time is an important measure. Existing research in HR-QOL outcomes in cardiac surgery demonstrate that long-term follow up is required to truly evaluate the impact of an intervention [11, 41, 42]. Seven studies measured the change in HR-QOL over time [14, 16, 23–25, 27, 29]. Only two included studies assessed the change in HR-QOL over the short and long term [14, 25]. Of mention, Endlich et al. prospectively measured postoperative MCS and PCS scores, providing valuable long-term data [14].

Three studies included in the current review measured preoperative scores in comparison to postoperative scores, which provides a valuable insight into the impact of surgery on these patients [16, 24, 29]. Of note, St Pierre et al. utilised SF36 and included over 100 patients in the final analysis [16]. Only four studies provide long term data (greater than 5 years postoperatively), highlighting the paucity of long-term outcomes [14, 15, 19, 25].

According to previous guidelines, a follow up rate of > 85% is considered ideal in systematic reviews [43]. None of the studies we evaluated attained this. This is understandable, as there was significant attrition due to mortality and morbidity associated with ATAAD surgery. This produces a selection bias; patients who do not participate in QOL assessment or those lost to follow-up potentially have worse QOL because of a greater burden of comorbidities, physical impairments, and psychological disturbance [44]. Studies with significantly low response rates are therefore more likely to skew the QOL results positively [44]. A number of studies had resultant small patient numbers [15, 17, 22, 24, 25]. Only four of the included studies incorporated greater than 100 patients [13, 16, 19, 21].

Well-designed prospective studies are required to make reliable conclusions on the HR-QOL outcomes after surgery for acute TAAD. These studies should utilise a standardised HR-QOL questionnaire such as SF-36 and make preoperative to postoperative comparisons along with long-term follow up. We appreciate that there are obstacles to this, as patients in the peri-operative setting are either unwell or the time critical nature of surgery prevents lengthy questionnaires. Furthermore, the mortality and morbidity of emergent ATAAD surgery produces attrition of follow up. As a result, the strength of evidence reviewed is limited.

## Summary of results and interpretation

HR-QOL outcomes are adversely affected in the postoperative period [14, 16, 24, 27, 29]. This result is not surprising, as ATAAD is associated with significant long-term morbidity. A recent study from the International Registry of Aortic Dissection found that 18% of patients had new renal insufficiency, 10% had new limb ischemia and 10% had major brain injury [45]. Four included studies assessed the change in HR-QOL over the operative period [16, 23, 24, 29]. Notably, three of these studies demonstrated that physical domains were significantly worse off postoperatively [16, 24, 29]. Studies that followed patients over the postoperative period also demonstrate impaired HR-QOL outcomes [14, 27]. Endlich et al. prospectively assessed 59 patients over the postoperative course [14]. The salient feature of this study was its long-term follow-up, which demonstrated significant attrition of both mental and physical health scores [14]. The loss of physical health over multiple time points provides a snapshot of the chronic course of the disease. Close to 20% of patients will require re-intervention within 5 years highlighting the chronic nature of the disease [46, 47]. Furthermore, a large portion of patients sustain a loss of function [48]. This takes an understandable toll on the physical health of patients and can account for the attrition of physical scores over time. Three studies suggest that mental health domains of patients, whilst initially affected, demonstrate some improvement over time. Notably, Sbarouni et al. demonstrated that that MCS scores at late time points are significantly higher than early timepoints [25]. Norton et al. demonstrated that 37% of patients reported severe anxiety in the postoperative period, reducing to 16% at late time points. These results reflect the emotional toll following emergency surgery and the adaptations patients make during the recovery phase.

Patients demonstrate worse HR-QOL outcomes following surgery when compared to age matched cohorts [13, 14, 18, 19, 26]. Four of the seven studies demonstrate significant impairment in physical domains after ATAAD repair [13, 14, 19, 26]. Two studies also demonstrate significant impairment in MCS scores [13, 14]. The largest of these studies was by Adam et al., which demonstrated that PCS scores were significantly lower than the norm sample across all age groups. This result is expected; ATAAD is an emergent disease and intervention is a life and death decision. Those that survive discharge out of hospital face challenges with recovery. Patients undergoing elective cardiac surgery on the other hand demonstrate a benefit in HRQOL over time. A systematic review of HR-QOL outcomes in aortic valve replacement demonstrate that the operative cohort do significantly better than age adjusted norm samples [11]. When considering aortic surgery, elective aortic surgery carries less risk than when procedures are done emergently [49]. As such, HR-QOL. outcomes in this setting fare well when compared to age matched cohorts [50]. This is consistent with other reviews investigating emergent aortic surgery. A systematic review by Shan et al. highlighted that the quality of life after emergent open abdominal aortic aneurysm repair was significantly worse than when the procedure is done electively and endoluminally [44].

Studies that assessed elderly cohorts of patients undergoing emergent surgery for ATAAD demonstrated attrition in HR-QOL outcomes postoperatively [13, 14, 20, 24, 29]. Of these included studies, two robust studies suggested that advancing age is associated with significantly worse PCSs postoperatively [13, 14]. The trend towards lower PCSs in the elderly is also demonstrated in other papers, albeit less robust [20, 24, 29]. Elderly patients are more likely to face major adverse cardiac and cerebrovascular sequalae after emergent surgery. One reason for this is that they are more vulnerable to the cerebral insult from deep hypothermic circulatory arrest which is required for some ATAAD repairs. As a result, the elderly cohort are more prone to lasting physical limitations compared to younger patients. Interestingly, two studies suggest that emotional well-being may be better in the elderly cohort compared to younger cohorts [13, 20]. While Endlich et al. demonstrated significantly lower MCSs in the elderly when compared to an age matched sample, they also demonstrated that younger patients have significantly worse MCSs by comparison [14]. For younger patients and their social environment, coping with the sequalae of an ATAAD is uncommon and more stressful. Younger patients often lose their job or require occupational training which is not the case for the elderly cohort. The combination of these may be attributable to the impact of MCSs, especially true in younger patients [14].

The sequelae of ATAAD poses an emotional toll on patients. This can lead to depressive disorders, PTSD and anxiety and are all linked with a loss of function [13, 21, 27]. Adam et al. demonstrated that one third of patients demonstrated symptoms of PTSD postoperatively and this was linked to diminished HR-QOL outcomes [13]. Luo et al. identified that sexual dysfunction was evident in 40% of patients postoperatively, with a significant impact on mental health in the younger population [21]. The survivors of ATAAD may benefit from psychological therapy in the postoperative setting, and those that are young or have been physically impaired as a result of the disease are particularly vulnerable.

The presence of preoperative neurological sequelae and malperfusion increases mortality and may negatively impact postoperative HRQOL. Bojko et al. demonstrated a significant association between malperfusion and mortality, which was most apparent in the elderly cohorts [15]. Only *Schachner *et al. reported the impact of malperfusion on postoperative HR-QOL, and found that patients with preoperative neurological symptoms and malperfusion had significantly lower postoperative activity [23].

Operative technique can affect the HR-QOL outcomes of patients with an ATAAD. The use of DHCA has been linked with poorer postoperative QOL in one study [28]. Findings suggest near normal HR-QOL postoperatively when DHCA is avoided or when ACP is used [26]. Distal anastomoses under DHCA may be favorable in certain circumstances, however DHCA is also associated with postoperative neurological sequalae and intraoperative coagulopathy, hence better HR-QOL when avoided. Larger prospective trials may validate this. The use of cerebral protection has been extensively studied from a mortality viewpoint, with multiple studies demonstrating a survival benefit from its use [51, 52]. Its translation to HR-QOL has not yet been validated; *Endlich *et al. did not demonstrate a significant QOL benefit from either perfusion strategy [14]. Ghazy et al. investigated the effects of an aggressive strategy with total arch replacement to a defensive strategy with ascending aorta replacement only and demonstrated longer operative times and potentially worse off physical function postoperatively [17]. Benefits of a defensive strategy include shorter cardiopulmonary bypass and circulatory arrest times, whereas an aggressive strategy may offer better long-term outcomes [17]. Short-term outcomes favour a defensive strategy, however there is still a paucity of long term data [17, 53]. Unless the clinical setting dictates an aggressive management strategy, a defensive strategy may be adopted with reasonable short-term HR-QOL outcomes [17].

### Limitations

We aimed at minimizing bias by reporting the strength of the study in terms of the number of patients included, design, comparison to preoperative status and rate of follow up. Smaller, retrospective studies with non-standardised HR-QOL outcomes were interpreted in light of larger, well-designed studies. The heterogeneity of literature with regards to HR-QOL measures, reporting and the demographics of the study population limited the role of meta-analyses.

### Future direction

Well designed prospective studies, with standardised HR-QOL outcomes such as SF12/SF36 utilizing preoperative and postoperative measurements are ideally suited to identifying the impact of ATAAD on quality of life. A further comparison to standardised HR-QOL measures would be useful. Long-term data provides useful insights into the chronic nature of the disease. Our review identified only four studies that assessed long-term outcomes, of these only one was prospective in nature. We also identified a paucity of research assessing operative strategy and its impact of HR-QOL outcomes. A further comparison of patients undergoing ascending aorta replacement only with an interposition graft, to a more aggressive strategy of total arch replacements and strategies of cerebral perfusion would be of great interest. We do acknowledge the emergent nature of the disease and the limited information that can be obtained in the preoperative setting as patients are often rushed to the operating theatre or are obtunded in the preoperative setting.

## Conclusion

HR-QOL outcomes are adversely affected when compared to preoperative status and physical health demonstrates significant attrition over time. HR-QOL outcomes are worse off when compared to an age matched general population. In terms of age, advancing age is associated with worse PCSs but emotional health may fare better than younger patients. To the authors’ knowledge, this is the only study to summarise HR-QOL literature in the setting of ATAAD. Well-designed prospective studies utilizing standardised HR-QOL measures would be of great value in drawing further conclusions.

## Supplementary Information

**Additional file1**: Table S1. Delphi quality appraisal tool. Table S2. Study characteristics. Table S3 Operative details and baseline patient characteristics.

## Data Availability

The datasets used and/or analysed during the current study are available from the corresponding author on reasonable request.

## Abbreviations

ATAAD: Acute Type A Aortic Dissection
HR-QOL: Health Related Quality of Life
PRIMSA: Preferred Reporting Items for Systematic Reviews and Meta-Analyses
MeSH: Medical Subject Headings
WHO: World Health Organization
SF36/SF12: Short Form 36/Short Form 12
PCS: Physical Component Scores
MCS: Mental Component Scores
PROMIS: Patient-Reported Outcomes Measurement Information System
PTSD: Post-traumatic stress disorder
DHCA: Deep Hypothermic Circulatory Arrest
