# Supplementary material for: Quality of life following surgical repair of acute type A aortic dissection: a systematic review
Source: J Cardiothorac Surg. 2022 May 16;17:118. doi: 10.1186/s13019-022-01875-x (PMC9112611; doi:10.1186/s13019-022-01875-x)
Supplement: Supplementary file 1 — Additional file1: Table S1. Delphi quality appraisal tool. Table S2. Study characteristics. Table S3 Operative details and baseline patient characteristics. [file 13019_2022_1875_MOESM1_ESM.docx]

| Supplementary Table 1: Delphi quality appraisal tool | |
| --- | --- |
| Criteria No. | Criterion definition |
| 1 | Is the hypothesis/aim/objective of the study stated in the abstract, introduction, or methods section? |
| 2 | Are the characteristics of the patients included in the study clearly described? |
| 3 | Were the cases collected in more than one center? |
| 4 | Are the eligibility criteria (inclusion and exclusion criteria) explicit and appropriate? |
| 5 | Were patients recruited consecutively? |
| 6 | Did patients enter the study at a similar point in the disease? |
| 7 | Did the authors describe the intervention? |
| 8 | In addition to intervention, did the patients receive any co-interventions? |
| 9 | Was loss to follow-up reported? |
| 10 | Are outcomes (primary, secondary) clearly defined in the introduction or methodology section? |
| 11 | Did the authors use accurate (standard, valid, reliable) objective methods to measure the outcomes? |
| 12 | Were outcomes assessed before and after intervention? |
| 13 | Was the length of follow-up clearly described/reported? |
| 14 | Were the statistical tests used to assess the primary outcomes appropriate? |
| 15 | Does the study provide estimates of the random variability in the data for the primary outcomes (e.g., standard error, standard deviation, confidence intervals)? |
| 16 | Was the analysis of outcomes based on intention to treat? |
| 17 | Are adverse events that may be a consequence of the intervention reported? |
| 18 | Are the conclusions of the study supported by results? |
| 19 | Is there a competing interest statement about the type and source of support received for the study or about the relationship of the author(s) or other contributors with the manufacturer of the technology? |

| **Supplementary Table 2: Study characteristics** | | | | | | | | | | | | |
| --- | --- | --- | --- | --- | --- | --- | --- | --- | --- | --- | --- | --- |
| Authors | Year | Country | Study Design | Total #patients | Demographic | Primary aim | Standardised HR-Qol measure? | Preoperative comparison? | Age and gender matched comparison? | Follow up timeframe | #patients followed up | Quality |
| Adam Et al^13^ | 2018 | Germany | R | 393 | Patients > 18 | Prevalence of PTSD and HR-QOL | Yes - SF12 | No | Yes | Mean - 51 months | 210 | Average |
| Endlich Et al^14^ | 2016 | Germany | P | 120 | Patients > 18 | HR-QOL | Yes-SF36 | No | Yes | 45 months, 90 months | 59 | Good |
| Bojko et al^15^ | 2020 | USA | R | 235 | Patients > 70 | Outcomes in elderly | Yes- SF36 | No | No | Mean - 75 months | 44 | Average |
| St Pierre^16^ | 2021 | USA | R | 369 | Patients > 18 | HR-QOL | Yes-SF36 | Yes | No | Within 36 months | 114 | Good |
| Ghazy et al^17^ | 2017 | Germany | P | 95 | Patients > 18 | HR-QOL with regards to operative technique | Yes-SF36 | No | No | 24 months | 39 | Average |
| Santini et al^18^ | 2006 | Italy | R | 40 | Patients > 75 | Outcomes in elderly | Yes-SF36 | No | Yes | Mean - 44 months | NR | Average |
| Jussli-melchers et al^19^ | 2016 | Germany | R | 242 | Patients > 18 | Outcomes in elderly | Yes-SF36 | No | Yes | 12 months | 167 | Average |
| Tang et al^20^ | 2013 | USA | R | 101 | Patients > 18 | Outcomes in elderly | Yes-SF36 | No | No | Mean - 17 months | 75 | Poor |
| Luo et al^21^ | 2010 | China | R | 204 | Patients > 18 | Sexual dysfunction postoperatively | Yes - SF12 | No | No | NR | 175 | Poor |
| Campbell-lloyd et al^22^ | 2010 |  | R | 65 | Patients > 18 | HR-QOL | Yes-EQ5D | No | No | Mean - 28 months | 29 | Poor |
| Schachner et al^23^ | 2019 | Austria | R | 131 | Patients > 18 | Physical activity | No | Yes | No | Median- 44 months | 91 | Poor |
| Vanhuyse et al^24^ | 2012 | France | R | 15 | Patients > 80 | Outcomes in elderly | Yes- WHO performance scale | Yes | No | NR | 15 | Poor |
| Sbarouni et al^25^ | 2021 | Greece | R | 45 | Patients > 18 | HR-QOL | Yes-SF36 | No | Yes | 12 months/60 months/120 months | 12 | Average |
| Immer et al^26^ | 2002 | Sweden | R | 107 | Patients > 18 | Effects of DHCA | Yes-SF36 | No | Yes | mean - 38 months | 69 | Average |
| Norton et al^27^ | 2021 | USA | P | 21 | Patients > 80 | Fitness following aortic surgery | Yes - PROMIS | No | No | 3 months/15 months | NR | Poor |
| Olsson et al ^28^ | 2013 | Sweden | R | 102 | Patients > 18 | HRQOL following surgery on proximal aorta | Yes-SF36 | No | Yes | mean - 40 months | NR | Average |
| Tashima et al ^29^ | 2020 | Japan | R | 103 | Patients > 18 | HR-QOL in elderly | No | Yes | No | Mean - 49 months | 67 | Poor |
| R, retrospective observational; P, prospective observational; HR-QoL, Health relate quality of life; | | | | | | | | | | | | |

|  |  | | | **Supplementary Table 3: Operative details and baseline patient characteristics** | | | | | | | | | |
| --- | --- | --- | --- | --- | --- | --- | --- | --- | --- | --- | --- | --- | --- |
| **Authors** | | **AscAo***  **Replacement** | **Bentall’s Procedure** | | **AoArch^**^ Replacement** | **Mean Age (years)** | **DHCA^***^, Degrees** | **Cerebral Perfusion Strategy %** | **Male** | **Smoker** | **Diabetes** | **Neurological deficit preoperatively** | **Malperfusion %** |
| Adam et al^13^ | | NR | NR | | NR | 59 | 559 (84%) | Retrograde 75%, Antegrade 6% | 132 (63%) | 61 (29%) | 6 (2.9%) | 72 (41%) | NR |
| Endlich et al^14^ | | 103 (86%) | 5 (4.2%) | | NR | 60 | 115 (96%) | Antegrade 25.8% | 84 (70%) | 69 (58%) | 16 (13%) | 33 (28%) | Total 28% |
| Bojko et al^15^ | | NR | 39 (16.6%) | | 15 (6.4%) | 77 | NR | NR | 102 (48%) | 51 (22%) | 38 (16%) | 24 (10%) | Total 23%, Mesenteric 7.5% |
| St Pierre et al^16^ | | NR | NR | | NR | 62 | NR | NR | 74 (65%) | 22 (19%) | 10 (8.8%) | NR | NR |
| Ghazy et al^17^ | | 26 (66%) | NR | | 13 (33%) | 61 | 95 (100%) 28^o^ | Antegrade 100% | 29 (74%) | NR | 4 (10%) | 5 (14%) | Total 23% |
| Santini et al^18^ | | 34 (85%) | 5 (12.5%) | | 11 (28%) | 78 | 40 (100%) 18^o^ | Antegrade 100% | 21 (53%) | NR | NR | 3 (7.5%) | Total NR, Limb 28% |
| Jussli-Melchers et al^19^ | | 203 (84%) | 39 (16%) | | 70 (29%) | 62 | 242 (100%) 18-22^o^ | Antegrade 47% | 154 (64%) | NR | 12 (5%) | 58 (24%) | Total 35%, Mesenteric 2.5% |
| Tang et al^20^ | | 71 (70%) | 22 (22%) | | 2 (2%) | 65 | 101 (100%) 22^o^ | NR | 63 (62%) | 19 (19%) | 12 (12%) | 9 (9%) | Total 49% (40% in < 80Y) |
| Luo et al^21^ | | NR | NR | | NR | 53 | 204 (100%) 25^o^ | Antegrade 100% | 148 (85%) | 119 (68%) | 29 (17%) | NR | NR |
| Campbell-lloyd et al^22^ | | NR | NR | | 9 (14%) | 61 | 34 (52%) | Retrograde 11%, Antegrade 46% | 39 (65%) | 36 (56%) | NR | NR | Total 57% |
| Schachner et al^23^ | | NR | NR | | NR | 59 | NR | NR | 67 (74%) | NR | NR | 16 (18%) | Total 31% |
| Vanhuyse et al^24^ | | 10 (60%) | 2 (13%) | | 2 (13%) | NR | 11 (53%) 28^o^ | Antegrade 26%, Retrograde 64% | 5 (33%) | NR | NR | 4 (27%) | Limb 7% |
| Sbarouni et al^25^ | | NR | NR | | NR | NR | NR | NR | NR | NR | NR | NR | NR |
| Immer et al^26^ | | 84 (79%) | 23 (22%) | | 0 | 62 | NR 20^o^ | NR | 74 (69%) | NR | NR | NR | NR |
| Norton et al^27^ | | 21 (100%) | 11 (52%) | | 0 | 55 | 21 (100%) | Antegrade 86%, Retrograde 4% | 18 (86%) | 12 (57%) | 1 (4.8%) | NR | NR |
| Olsson et al ^28^ | | 95 (93%) | NR | | 7 (7%) | 57 | NR | NR | 145 (70%) | 65 (31%) | 5 (2%) | NR | NR |
| Tashima et al ^29^ | | 79 (77%) | 3 (3%) | | 21 (20%) | 70 | 103 (100%) 20-26^o^ | NR | 46 (45%) | NR | 5 (5%) | 6 (6%) | Total 18%, Mesenteric 7% |
| *AscAo: Ascending aorta  **AoArch: Aortic arch  ***DHCA: Deep hypothermic circulatory arrest  NR: Not reported | | | | | | | | | | | | | |
|  |  | | |  | | | | | | | | | |
